# Studying the Effects of Dissolved Noble Gases and High Hydrostatic Pressure on the Spherical DOPC Bilayer Using Molecular Dynamic Simulations

**DOI:** 10.3390/membranes14040089

**Published:** 2024-04-12

**Authors:** Eugeny Pavlyuk, Irena Yungerman, Alice Bliznyuk, Yevgeny Moskovitz

**Affiliations:** 1 Laboratory of Multi-Scale Mathematical Modeling, Ural Federal University, Ekaterinburg 620002, Russia; 2 Department of Chemical Engineering, Technion—Israel Technological Institute, Technion City, Haifa 3200003, Israel; 3 Ilse Katz Institute for Nanoscale Science and Technology (IKI), Ben Gurion University of the Negev, Beer Sheva 8410501, Israel

**Keywords:** liposome, molecular dynamics, inert gas narcosis, high-pressure neurological syndrome

## Abstract

Fine-grained molecular dynamics simulations have been conducted to depict lipid objects enclosed in water and interacting with a series of noble gases dissolved in the medium. The simple point-charge (SPC) water system, featuring a boundary composed of 1,2-Dioleoyl-sn-glycero-3-phosphocholine (DOPC) molecules, maintained stability throughout the simulation under standard conditions. This allowed for the accurate modeling of the effects of hydrostatic pressure at an ambient pressure of 25 bar. The chosen pressure references the 240 m depth of seawater: the horizon frequently used by commercial divers, who comprise the primary patient population of the neurological complication of inert gas narcosis and the consequences of high-pressure neurological syndrome. To quantify and validate the neurological effects of noble gases and discriminate them from high hydrostatic pressure, we reduced the dissolved gas molar concentration to 1.5%, three times smaller than what we previously tested for the planar bilayer (3.5%). The nucleation and growth of xenon, argon and neon nanobubbles proved consistent with the data from the planar bilayer simulations. On the other hand, hyperbaric helium induces only a residual distorting effect on the liposome, with no significant condensed gas fraction observed within the hydrophobic core. The bubbles were distributed over a large volume—both in the bulk solvent and in the lipid phase—thereby causing substantial membrane distortion. This finding serves as evidence of the validity of the multisite distortion hypothesis for the neurological effect of inert gases at high pressure.

## 1. Introduction

A hyperbaric aquatic environment provides an ideal setting for a comparative examination of hyper- and hypoexcitability processes within the central nervous system (CNS) of living organisms, offering a unified framework for research. The opposite neurological conditions emerge distinctly, influenced by both the compression factor and the water horizon, where compromised signal transduction processes are explored. The specific type of life-endangering hypoexcitability is referred to as inert gas narcosis (IGN) when the inert noble gases are used in the breathing mixture [1,2]. Alternatively, it is termed nitrogen narcosis when hyperbaric air is inhaled by the immersed diver or hyperbaric chamber personnel in a pressure above 4 bar [3]. Conversely, commercial divers exposed to the lightest noble gas’s—helium’s—ambient pressures above 11 bar are susceptible to hyperbaric hyperexcitability, or so-called high-pressure neurological syndrome (HPNS), which is phenomenologically reciprocal to IGN and associated with tremors, myoclonus and convulsions [4,5]. 

According to the most general definition, anesthesia is induced by volatile hydrophobic molecules that can percolate the blood–brain barrier and disrupt routine chemoelectrical signal transduction in the brain neurons [6,7]. Aside from the broad class of potentially harmful anesthetic molecules used in clinical practice, noble gases have the simplest chemical structure; furthermore, their narcotic potential can be triggered only in a hyperbaric medium, as the concentration of dissolved gas increases with hydrostatic pressure. There is one exception—large heavy polarizable xenon atoms may be applied as a powerful anesthetic even under standard conditions [8,9]. The theoretical foundation falls behind the outstanding empirical success of anesthetic substances; there has yet to be a broad consensus on the molecular mechanism of general anesthetic (GA) action [10]. The latest fundamental theories analyzing narcosis stem from the late 19th century when the Meyer–Overton rule was formulated; this rule states that anesthetic potential depends on molecule solubility in the lipid phase [11]. Given the inability of many hydrophobic compounds to comply with this statement, for instance—chirality must be further considered [12]; the modern protein theory has emerged posing membrane-embedded proteins (TMs) as the primary target of anesthetic action [13]. The neuroanatomic data imply that various neuron populations possess different types of TM channels and lipid membrane chemical composition—lipidome—hence, neurons react non-uniformly to intervene anesthetic molecules [14]. The dominant brain areas affected by general anesthesia are located mostly in the forebrain which plays a critical role in maintaining the state of consciousness [15]. 

High hydrostatic pressure per se applied on anesthetized organisms above 100 bar reverses deep CNS sedation by pharmacological agents [16]. Uniform hydraulic compression evoked tremors, uncoordinated limb movements, and tonic convulsions in unanesthetized liquid-breathing mice in pressures ranging from 50.6 to 101.3 bar [17]. Shallow-water animals performing extremely deep dives may be exposed to hyperexcitability, whereas deep-sea evolutionary inhabitants are well adapted to high pressure (HP) [18]. It is yet to be determined at what critical threshold HP may be self-sufficient in provoking HPNS and the extent to which hydrostatic pressure overcomes the effects of dissolved hyperbaric helium at the molecular/cellular level. Although HP is the most common thermodynamic factor, its effects on the CNS unexpectedly exhibit strong localization patterns. Visualizations of brain regions under HP have been conducted using voltage-sensitive dyes, while networks of up to 108 neurons in a rat hippocampal slice have shown irritation at 32 bar [19]. These data align with the hypotheses of protein dominance also in governing neurological response to HP; protein conformations indeed reveal extreme sensitivity to the slightest fluctuations in temperature or pressure [20]. However, there is no unequivocal evidence to extend the protein theory of narcosis to the neurological effects of anesthetic noble gases compared to the effect of helium, which acts as a pressure transducer. This is due to the absence of clear evidence regarding the gases’ preferred interaction with extramembrane protein domains. Contrary, the noble gases’ solubility in the lipid phase is well known and directly correlates with the gases’ atomic weight. According to these properties, the anesthetic potency decreases in the following order (with the atomic number displayed in parentheses): Xe(54) > Kr(36) > Ar(18) > Ne(10) > He(2). The physiological data indicate that, in addition to xenon, krypton and argon are strong anesthetics in P > 2 bar; neon’s narcotic and excitative potency is poorly known; helium has no reported narcotic tendency—at least in physiologically reasonable pressure ranges, up to 100 bar [21]. 

Lipids’ structural and dynamical parameters under the influence of GA and HP have been intensively studied by experimental and theoretical methods [22,23]. Computer experiments combining the advantages of both approaches are proven as especially promising since they provide an unprecedented level of resolution at the nanometric scale. The clarity of the molecular simulation outputs directly depends on the quality of the force field (FF) applied related to its ability to reproduce experimental data. The most recent advances in biomolecular force field refinement have been implemented in a series of optimizations of force field (FF) parameters [24,25]. In addition to FF settings, the basic geometry of the system under investigation can also affect the output data [26]. Molecular dynamics (MD) simulations have been employed to model planar lipid bilayers in cubic boxes with periodic boundary conditions (PBCs), assuming the lateral homogeneity of the cell membrane. The volume of the water phase is considered to be equal to or even smaller than the lipid phase volume. Under these conditions, the potential influence of noble gas atoms on lipid bilayer distortion, which could be a factor in the neurological responses, has been explored in previous research [27,28]. It was concluded that while in moderately low hydrostatic pressure per se (P < 100 bar) very few structural changes have been observed, the fraction of dissolved noble gas causes substantial distortions of lipids’ packing order. The large atoms of xenon tend to increase the area per lipid upon their percolation to the lipid phase resulting in a lateral expansion of the membrane. The smaller gas atoms, like neon, on the other hand, tend to increase the bilayer thickness along the normal to bilayer plane [29,30]. 

The intrinsic limitation of the lipid bilayer lateral homogeneity approach and the neglect of the processes taking place in the bulk solution make it impossible to follow up the conjunction of the molecular events occurring at the extended boundary of two phases. The abovementioned simulations could therefore be biased toward the statistically less probable thermodynamic outcome. For instance, particular attention should be given to the dispersed gas fraction’s propensity to aggregate and form nanobubbles. While gas nanobubbles in the bulk solvent near a hydrophobic substrate are traceable through experimental methods, accurately reproducing them in simulations with a limited solvent capacity might be challenging [31]. In contrast, the distortion of a planar bilayer caused by a continuous interlayer bubble of hyperbaric helium, observed in simulations, might be overestimated when compared to helium diffusion into the bilayer with finite closedness [32]. The concept of finite bilayer closedness pertains not to the molecular scale but rather to the subcellular scale of biological signal transduction cascades, as exemplified by the molecular machinery of the synaptic vesicle (SV) release. GA is known to depress SV exocytosis [33]; similarly, the high hydrostatic pressure slows down the vesicle fusion process [34]. The overall synaptic transmission blockade by GA vs. high hydrostatic pressure and hyperbaric helium, however, results in opposite outcomes with different excitatory/inhibitory ratios of molecular signals. Research with the atomistic resolution of impaired SV release is essential for the elucidation of the key mechanisms underlying synaptic communication. The enclosed bilayer or liposome simulations are increasingly important i.a. due to the rise of drug nanocarriers for biomedical use [35]. The limits of liposomal stability and loading protocols require substantial clarification. Following the above, we suggest a liposome model immersed in the massive bulk solvent for further IGN/HPNS processes’ refinement.

## 2. Methods

The enclosed spherical bilayer composed of 1683 1,2-Dioleoyl-sn-glycero-3-phosphocholine (DOPC) molecules in a cubic box pre-equilibrated in the planar bilayer molecular dynamics with a GROMOS87-based force field was solvated with 352,928 water molecules as a starting point [36]. The GROMOS87-based FF model we used has been recently meticulously tested for satisfactory appropriateness in the study of xenon anesthesia in a planar fully hydrated di-palmitoyl-phosphatidyl-choline (DPPC) membrane and has been compared against the CHARMM36 FF model [37]. The bilayer density profiles, lipid tails’ order parameters, lateral diffusion coefficients and energy profile of the headgroup barriers have been determined as tolerably similar. The Packmol 16.143 script was used to position the DOPC lipids along the radius of the liposome [38]. The external monosphere of the liposome contained 1188 DOPC molecules vs. 495 in the internal monosphere. The concentration of the DOPC molecules was as follows: n = N/S; S—the area of the liposome sphere, N—the amount of DOPC molecules. n was selected as the reference molecular density of the planar bilayer for both monospheres [29]. 

The extended simple point-charge (SPC) water model was used, and periodic boundary conditions (PBCs) were employed. In all the deployed systems exposed to high pressure, the gas concentration was constant to ascertain pure pressure effects, and a completely saturated bilayer was assumed; 5513 bulk solvent molecules were randomly replaced by gas atoms, assuming the 1.5% molar concentration of dissolved gas. The bilayer exposed to pure solvent was tested as a point of reference. The gas atoms were treated as simple Lennard-Jones (LJ) sites with the following interaction parameters: for xenon, ε = 1.900 kJ mol^−1^ and σ = 0.406 nm; for argon, ε = 0.979 kJ mol^−1^ and σ = 0.340 nm; for neon, ε = 0.289 kJ mol^−1^ and σ = 0.278 nm; and for helium, ε = 0.084 kJ mol^−1^ and σ = 0.256 nm. The same parameters were used in previous simulations of gases in lipid bilayers [29,32]. All simulation systems were energy-minimized by the steepest descent method for 10^4^ steps, with NVT and NPT-coupled pre-equilibration for 1 ns before submitting the data to a production run. 

The GROMACS 5.0.2 package was used in the simulation setup and analysis [39]. The Lomonosov supercomputer at Moscow State University’s HPC facility was used in our calculations. Each production simulation lasted for at least 100 ns (divided into 1 ns sampling subtrajectories). The pressures we tested included 1 bar (reference) and 25 bar at a constant temperature of 310 K determined for the gel–crystalline phase in previous studies, where 25 bar is considered as practically relevant for both IGN and HPNS outsets. The Berendsen method was used to maintain the pressure–temperature coupling, while bulk solvent and bilayers were controlled separately with a relaxation time of 0.25 ps; the gas atoms were coupled to the solvent phase. The Berendsen weak coupling method was applied in the planar bilayer control simulation [29]. It proved very efficient in equilibrating the simulation system. It does not sample the exact NPT statistical ensemble and may lead to artifacts in aqueous biopolymers or liquid/liquid interfaces [40]. However, as the number of particles increases, the Berendsen method becomes more similar to canonical ensemble sampling. Isotropic pressure settings at 1 bar and argon molar concentrations of 0.375%, 0.75% and 1.5% have been tested for 50 ns to determine the minimal concentration threshold of the bubble nucleation process. The isotropic pressure control has also been compared with semi-isotropic at 25 bar. A time-step leapfrog integrator was used every 2 fs, and the linear constraint solver (LINCS) algorithm was applied to preserve the bond lengths. The non-bonded pair list was updated every 10 steps with a cutoff of 1.0 nm. For the short-range van der Waals interactions, a cutoff distance of 1.0 nm was used. The long-range electrostatic interactions were treated by the particle mesh Ewald method with a grid spacing of 0.12 nm; cubic interpolation was adopted in these instances. A volume compressibility of 4.5 × 10^−5^ bar^−1^ was selected. Data were collected every 2 ps.

Python scripts were written for statistical data analysis, applying GROMACS analytical utilities on MD trajectories at a frequency of 1 ns^−1^ to ensure the sufficient sampling of statistically independent data. The modified GROMACS 4.6.7 version for the DOPC order parameter evaluation was employed [41]. The LAMMPS package (released 08/2019) analysis tools for spherical coordinates were used for the DOPC/gas density calculation [42], while the original Python 3 code was used to evaluate the distribution of gas atoms along the radius of the liposomes considering gas–solvent/DOPC atoms’ contact cutoff of 1.0 nm to account for adjoined gas. The ^3^V server was used to assess the molecular volume data [43]. Finally, Pymol 2.3.4 was used for the 3D presentation of representative MD frames [44]. 

## 3. Results

**Figure 1:**
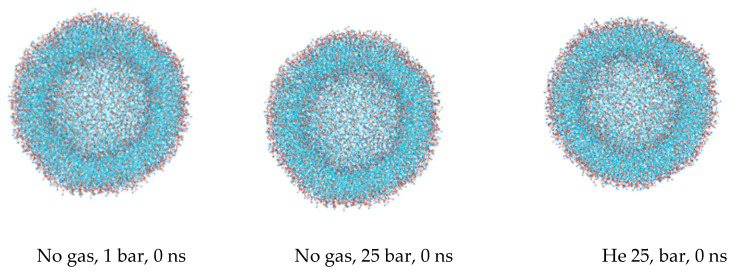

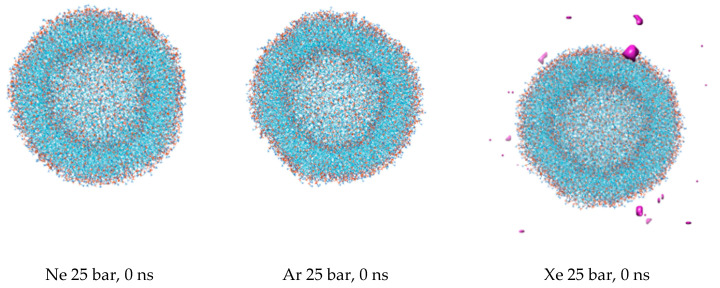
The initial frames of the liposome at the beginning of the molecular dynamics production cycle at t = 0 ns are shown for 6 simulations conducted at 25 bar of ambient pressure. The small droplets of xenon in the bulk solvent are designated by the violet color.

**Figure 2:**
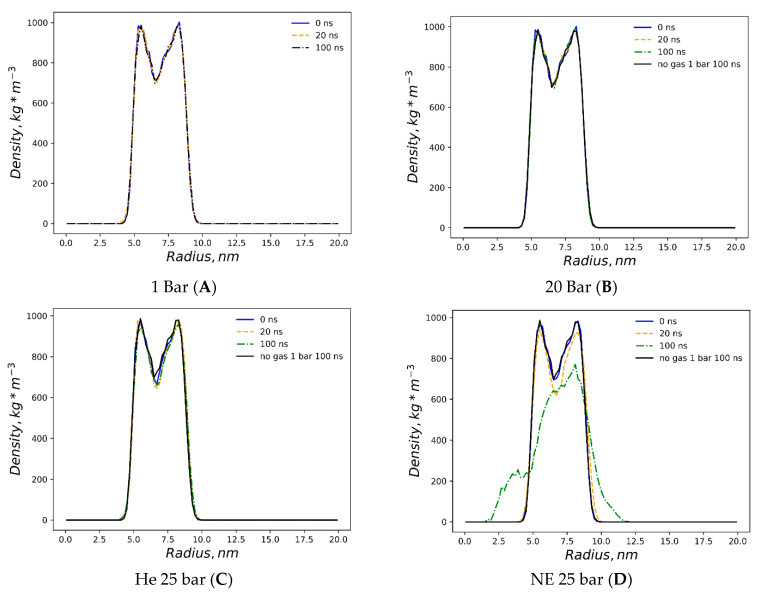

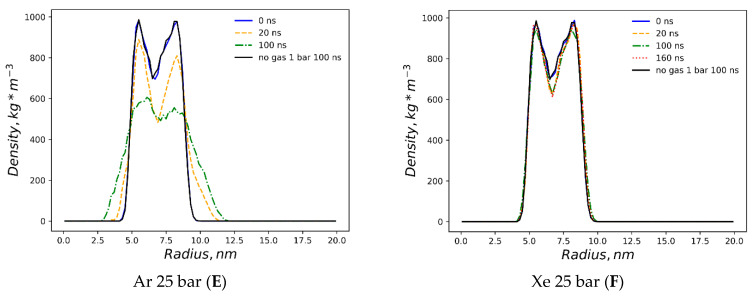
The density (kg·m^−3^) of DOPC lipids (*y*-axis of the plots) is presented for 6 simulations at 0, 20, 100 and 160 ns. The *x*-axis is the radius of the liposome (nm) centered at the center of mass (x = 0) comprising DOPC molecules. (**A**) 1 bar, (**B**) 25 bar, (**C**) He 25 bar, (**D**) Ne 25 bar, (**E**) Ar 25 bar, and (**F**) Xe 25 bar.

**Figure 3:**
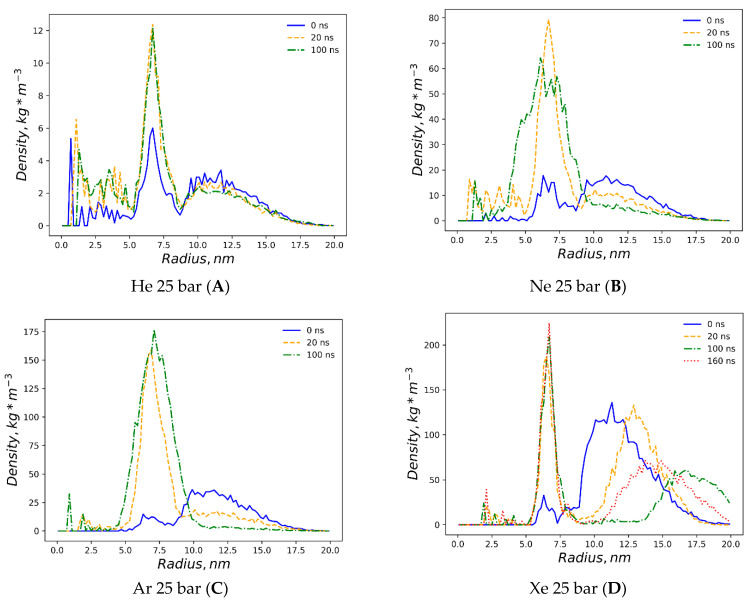
The density (kg·m^−3^) of noble gas atoms (*y*-axis of the plots) is presented for 4 simulations at 0, 20, 100 and 160 ns. The *x*-axis is the radius of the liposome (nm) centered at the center of mass (x = 0) comprising DOPC molecules. (**A**) He 25 bar, (**B**) Ne 25 bar, (**C**) Ar 25 bar, and (**D**) Xe 25 bar.

The first snapshots of the liposome at the outset of each simulation are displayed in Figure 1. The preliminary equilibration procedures with dissolved gas fractions in the bulk solvent do not introduce detectable distortions to the liposome’s shape. The DOPC density presented in Figure 2 is a collection of local densities at spherical bins, with a thickness of 0.2 nm spread out along a 0.0–20.0 nm range from the center of the mass of the liposome. The data presented encompass several MD frames at 0, 20 and 100 ns. The data in ref [29] correspond to the referential average densities of the stacked DOPC atoms assigned as bilayer thickness profiles for the planar bilayer. The DOPC density of the native liposome with isotropic pressure settings under standard conditions is presented in Supplementary Figure S1A. The semi-isotropic pressure referring to the bulk solvent compression characteristic of the planar bilayer has been further suggested as a work model providing the data listed below. The undisturbed liposome with no gas under standard conditions (Figure 2A) demonstrates the visible skewness of the enclosed spherical packing of DOPC molecules, while the outer liposome monosphere is not a mirror image of the inner monosphere relative to the point of the lipids’ density interlayer minima; it extends to 0–3 nm vs. 0–2.5 nm at the planar bilayer. Despite this skewness, the headgroup densities represent peak values close to the 1000 kg m^−3^ typical of the native planar bilayer. Along the entire 100 ns course of production, the native liposome preserves its characteristic density profile with no statistically significant fluctuations observed. The increase in the hydrostatic pressure value to 25 bar (Figure 2B) does not introduce any changes in the distribution of the packing density of lipid molecules in spherical monolayers, while the stability of the density profile in the range of 100 ns coincides with that of the native liposome under standard conditions. The disruption of the DOPC density by helium atoms at 25 bar is presented in Figure 2C, while the referential density values for the planar DOPC membrane distorted by helium at 3.5% molar concentration is presented in ref. [32]. Unlike the massive planar bilayer distortion by a homogeneous ovoid-shaped helium microbubble, the liposome with a 1.5% molar concentration of dissolved helium does not undergo bilayer inflation and therefore maintains its native density profile boundary. As predicted, however, the extended helium bubble formed in the gap between spherical monolayers; this is presented in the gas density plots of Figure 3A. The helium density complex profile emerges right after the preliminary NPT equilibration, while helium atoms uniformly distributed throughout the bulk solvent tend to diffuse toward the hydrophobic liposome core and create a smooth, increasing wave-like helium density peak adjacent to the outer monolayer of the liposome. The intra-bilayer helium’s sharp density peak stabilized after a few ns and remains at 12 kg m^−3^. The dissolved helium phase proved capable of fully percolating the lipids and dissipating into the inner confined polar solvent, presenting variable density peaks of 2–6 kg m^−3^. The liposome’s density incurs irreversible deformation under neon at 25 bar (Figure 2D); following the initial uniformly extended neon bubble formation in the hydrophobic gap at 0–20 ns, the asymmetric native density profile breakup occurs at t > 20 ns. The neon density shown in Figure 3B presents the incipient accumulation within the inner liposome monosphere.

The dissolved argon’s effects on the native liposome under standard conditions at 1 bar with isotropic pressure settings are presented in Supplementary Figure S1A,B, while a molar concentration of up to 0.375% has been shown as having a minimal distortion of the DOPC profile. The argon effects with increased molar concentration 1.5% and isotropic pressure at 25 bar are depicted in Figure S2A,B. The very similar density profile of the DOPC phase under the influence of hyperbaric dissolved argon at a semi-isotropic pressure of 25 bar is depicted in Figure 2E. As with neon, and in contrast to helium at the same pressure, the filling of the hydrophobic volume with argon atoms occurs in several steps, resulting in the significant destruction of the original DOPC density profile. Unlike hyperbaric neon, argon deformation causes an even more pronounced rarefaction of the DOPC density in the hydrophobic core; the numerical value in the center of the lipid phase decreases drastically below 600 kg m^−3^, while the average density profile stays rather symmetrical, although it shifted toward the inner liposome monosphere similar to neon distribution. However, unlike helium and neon, the argon gas density (Figure 3C and Figure S2B) presents its almost complete accumulation within the liposome contour, with a few peaks appearing in the confined solvent and no peaks traced at the bulk phase at 100 ns.

The simulation with hyperbaric xenon at 25 bar has been extended beyond 100 ns due to its atomic mass characteristics, with the contour of the DOPC density at 0–160 ns is presented in Figure 2F. The DOPC density pattern at t > 20 ns closely resembles helium distortion (Figure 2C) without breaking the packing order of polar headgroups. The hydrophobic cleft of the liposome deepens upon xenon loading in comparison to inflation by helium, with the DOPC density at the cleft as follows: (Xe) 600 vs. 660 kg m^−3^ (He). The xenon atom’s density distribution is shown in Figure 3D. Unlike the other dissolved gas atom populations, the xenon-dissolved fraction is clearly separated into two subphases following a production MD run of less than 20 ns. The first fraction occupies the hydrophobic core of the liposome and remains unchanged for 160 ns, while the second fraction is an isolated region in the bulk solution. Diffusing away from the liposome’s center of mass for 20–100 ns, the initial distancing, subsequent density redistribution and the approach of the compact-isolated xenon phase to the lipids last for 100–160 ns. No contact is restored between the two xenon phases by the end of the simulation.

**Figure 4:**
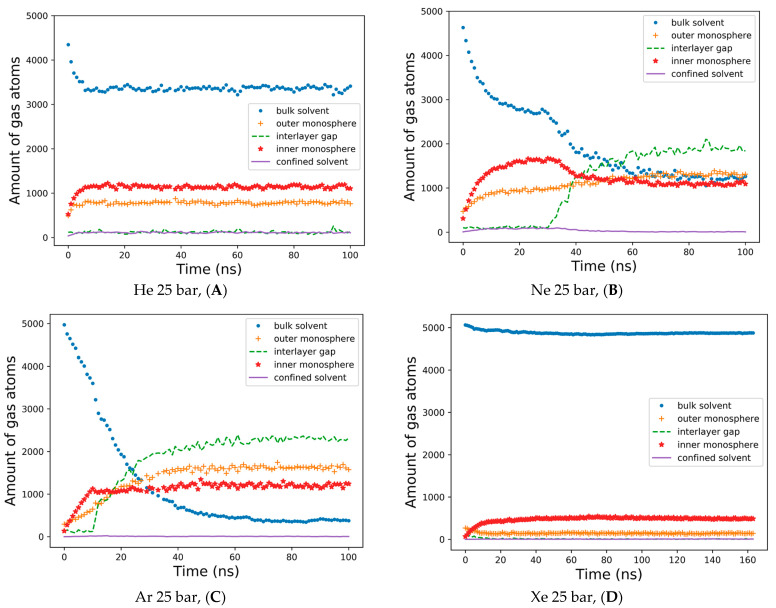
The dynamics of gas percolations in time. The number of effective contacts is shown between gas atoms and the bulk solvent; DOPC atoms in the outer and inner monolayers of the liposome; the interlayer gap and confined solvent molecules. The contacts’ sampling frequency—1ns. A contact cutoff of 1.0 nm has been applied. (**A**) He 25 bar, (**B**) Ne 25 bar, (**C**) Ar 25 bar, and (**D**) Xe 25 bar.

The detailed dynamics of gas percolations shown in Figure 4 reflect the consideration of the five spatial domains in the simulation box for tracking the number of gas atoms vs. the MD time. The gas atoms were counted at every integer nanosecond MD frame, applying a cutoff of 1.0 nm to referential heavy atoms in the external bulk solvent, the outer lipid monolayer and the interlayer gap—defined by the terminal carbons of each monolayer, inner monolayer and confined solvent. The helium percolation dynamics are presented in Figure 4A. Following the fast lipid saturation for the first 10 ns of production MD, 70% of dissolved helium atoms remain in the bulk solvent—contrary to the previously reported case study of the planar bilayer [32]. More than 20% of helium fractions populate the inner monolayer, while 15–16% are allocated to the outer monolayer, and less than 5% are equally distributed between the monolayer gap and the confined solvent. Unlike helium and in conjunction with the previous report [29], the neon atoms showcase the tendency to fill up and inflate the interlayer gap, while the neon bulk fraction drops to 30%, and the fraction in the gap rises to 40% after 50 ns (Figure 4B). Under neon, the inner monosphere shows oversaturation in the first 40 ns, followed by a gas release before reaching a steady state with the outer monosphere at approximately 30%. A limited percentage of neon atoms stay entrapped within the confined solvent at this stage.

The interlayer bubble’s formation is maximized with argon. The argon fraction of atoms in the interlayer space is approximately 50%, while the argon fraction in the bulk solution decays exponentially to 10% by the end of the simulation (Figure 4C). Unlike helium and neon, argon atoms mostly populate the outer monosphere of the liposome, although their saturation is slower than in the inner monosphere: representing 30% vs. 24% of the total argon fraction in each lipid monosphere, respectively. The argon fraction of the confined solvent remains similar, at a level of 1–3% from the total amount of gas atoms. Supplementary Figure S3 presents a very similar pattern for the dynamics of hyperbaric argon’s percolation under isotropic pressure settings. The distribution of xenon for a 160 ns simulation is shown in Figure 4D. During the entire course of the simulation, more than 90% of the heavy xenon atoms flock to the external bulk solvent, as presented in Figure 3D. The interlayer inflation of the liposome under hyperbaric xenon is negligible, while the fraction of xenon atoms in the gap accounts for less than 1%. The total xenon atoms entrapped in the lipid phase were 7–8% in the inner monolayer vs. 2–3% in the outer monolayer.

**Figure 5:**
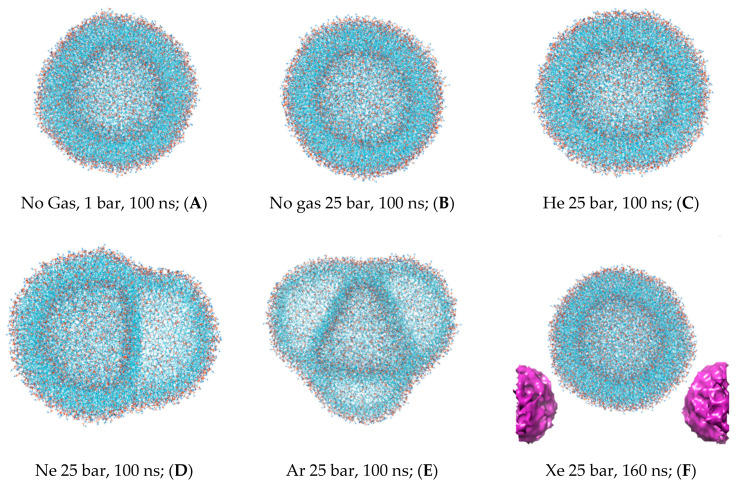
The representative last frames of the liposome trajectory for 6 simulations. (**A**) 1 bar, (**B**) 25 bar, (**C**) He 25 bar, (**D**) Ne 25 bar, (**E**) Ar 25 bar, and (**F**) Xe 25 bar. The xenon free-floating bubble in periodic boundary conditions is presented in a violet color.

The data analyzed in Figure 4 enabled us to determine a trait MD range for the redistribution of various gas phases—from random uniform spreading in the bulk solvent to specific hydrophobic compartment infiltration and gas atom entanglement in the structural units of the DOPC liposome. We determined that an MD time of 20–60 ns is typical for accomplishing phase transitions between deferent compartments in the box; assuming that the simulated system reaches a steady-state condition by the end of each >100 ns simulation, we selected the final frame of every MD trajectory for 3D visualization and molecular volume processing. Thus, the isolated DOPC molecular coordinates are shown in Figure 5A–F for all six experiments. Figure 5F includes additional free-floating single xenon bubble coordinates in PBC. The intermediate liposome frames are also available in Supplementary Material Figures S4–S9.

The representative molecular frames of the liposome have been uploaded to the ^3^V server. The virtual probe of radius 1.4 Å, which approximates the radius of a water molecule, was used to present the liposome shape parameter evaluation (Table 1). The control simulation data under standard conditions with no gas were used as a normalization parameter (upper row), while the hyperbaric experiment data are presented as a dimensionless score related to the control simulation. The effective radius of the undisturbed liposome in the control simulation, 3.76 nm, primarily refers to the inner monosphere. This is because of the expanded area per lipid in the outer monosphere and is affected by the fixed 1.4 Å solvent probe radius used to generate the molecular surface. However, it is important to note that this assumption presupposes that water molecules approach all surface atoms at an equal distance regardless of polarity, which does not accurately reflect the conditions in our simulations.

Primarily, the native spherical liposome—as presented in Figure 5A—shows insignificant intrinsic lipid molecular volume variations (less than 1%, presented in the first left-hand column of Table 1). All explored noble gases increase the total molecular volume of the liposome. The increase is the most prominent with argon atoms, while powerful anesthetic xenon enlarges the liposome volume to a lesser extent than non-anesthetic neon and to a greater extent than helium. The hydrostatic pressure (Figure 5B), on the other hand, slightly decreases both the molecular volume and total surface area of the liposome (as shown in the second column of Table 2). However, apart from helium, the extent to which gases enlarge the surface area is not identical to the volume increase. Asymmetrical deformations of the liposome via interlayer bubbles of neon and argon (Figure 5E,D) are strikingly distinctive from helium and xenon distortions (Figure 5C,F); the bubble formation causes overstretching and results in a substantial increase in the surface area up to 1.7-fold with argon simulation. The liposome’s stretching is additionally expressed in its sphericity index score and dimensionless radius (as shown in the third and fourth columns of Table 1): 0.6 and 0.76 for argon and neon, respectively.

**Figure 6:**
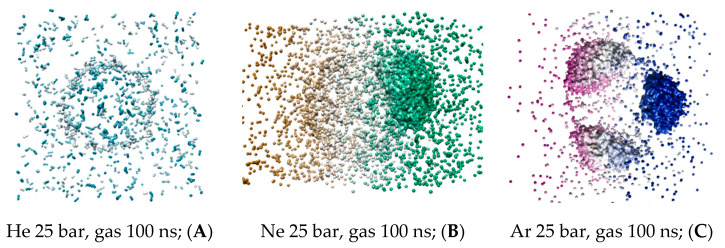
The representative last frames of MD trajectory: (**A**) helium 25 bar; (**B**) neon 25 bar; and (**C**) argon 25 bar. The continuous gas fractions are depicted by the ^3^V server using probe radius 6.0 Å [30]. The color spectrum represents the three-dimensionality of the molecular volumes.

The coordinates of gas atoms from the last trajectory frame have been processed and visualized on the ^3^V server, as shown in Figure 6A–C. The virtual probe of radius 1.4 Å was used in a similar manner to the DOPC molecular volume visualization. The virtual probe does not display individual atoms but encompasses the condensed gas phase collected in droplets larger than a single water molecule. Intermediate helium, neon and argon MD frames at 20–40 ns are available in Supplementary Figures S6–S8. The fusion of non-bound xenon microbubbles is shown in Figure S9.

To analyze the molecular volume of all the condensed gas fractions presented in Table 2, the condensed gas fraction associated with the bulk solvent was used as a normalization parameter (shown in the first left-hand column) for the fractions associated with the liposome—presented in dimensionless volumes. Unlike the atoms’ counter in Figure 4A,B, the helium and neon condensed bulk solvent fractions have similar capacities. However, neon adsorbed fractions within the lipid phase are significantly larger than the helium condensed fraction. Argon’s capacity in the interlayer gap exceeded its condensed volume in the bulk solution by almost 15-fold, while heavy xenon atoms formed no detectible condensed phase in the liposome. This implies that the amount reported in Figure 4D is distributed as scattered loosely particles within the inner DOPC monolayer rather than a continuous xenon bubble.

**Figure 7:**
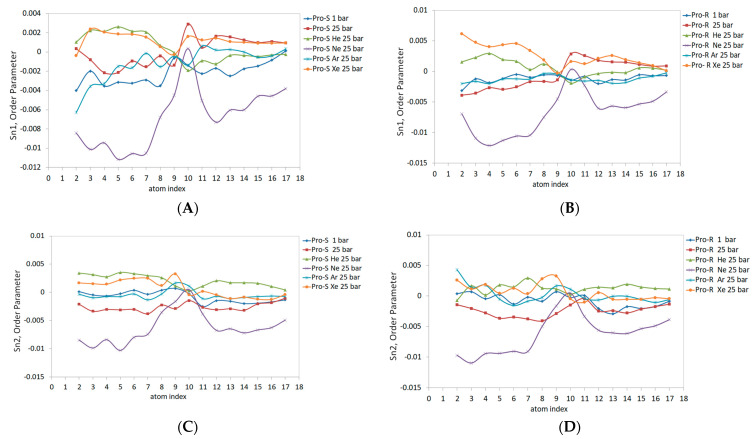
External monosphere DOPC acyl chains Sn1 and Sn2 prochiral order parameters: (**A**)—Sn1: Pro-S; (**B**)—Sn1: Pro-R; (**C**)—Sn2: Pro-S; and (**D**)—Sn2: Pro-R.

The basic lipid packing arrangement in the liposome is defined as the frustum shape. This is the fundamental geometrical subunit composed of the lipid molecules of both monolayers. The varying area per lipid along the liposome radius ranges from 0.95 nm^2^ at the outer surface of the external monolayer to 0.5 nm^2^ at the inner surface of the internal monolayer. It is worth noting that the direction for the area per lipid in the outer monolayer is opposite that of the inner monolayer. This is principally different from the constant 0.6–0.7 nm^2^ area per lipid in the planar bilayer, given the cylindrical packing geometry of this area. Considering the non-constant area per lipid, the DOPC order parameters have been analyzed independently for the Sn1 and Sn2 acyl chains in each spherical monolayer. The last 20 ns of each MD trajectory were sampled for DOPC order parameter evaluation. Figure 7 illustrates the order parameter data in molecular prochiral frames Pro-S and Pro-R for the outer monolayer of the liposome. The rationale for separating the order parameter of acyl chains into Pro-S and Pro-R order parameters is explained in ref. [41]. The same parameters for the planar DOPC bilayer are presented in ref. [32]. Compared to the planar bilayer data, the outer monolayer data reveal a markedly lower Sn1 magnitude (Figure 7A,B), especially under hyperbaric neon—Sn1 is negative, also presenting negative concavity and peaking only at double-bond (C10) carbons up to 0. The Pro-S and Pro-R curves also differ to a larger extent than previously reported; for example, the effects of xenon and helium were overestimated relative to the 1 bar control line for both Pro-R and Pro-S in the C1-C9 region. Conversely, the hydrostatic pressure at 25 bar slightly underestimates the order parameter in C1-C9 in the Pro-R approach and overestimates the Pro-S line in the same region. The argon curve in C4-C17 extends above the control line at Pro-S. Considering both Pro-S and Pro-R approaches, the pressure exhibits an elevated curve in the C10-C17 range (Figure 7A,B). Xenon and helium also overestimate the C10-C17 region of Sn1 at Pro-S and Pro-R frames; however, the Pro-R argon 25 bar curve almost unites with the control line while simultaneously rising above the helium curve at Pro-S.

The Sn2 data for both prochiral molecular frames are presented in Figure 7C,D. The data are generally characterized by lesser Pro-S/Pro-R differences resembling the planar bilayer pattern. The Ne 25 bar line and pressure at 25 bar have the lowest negative Sn2 values, with a maximum of 0 at C10-C11 carbons, while heavy xenon and argon atoms enlarge Sn2 Pro-S and Pro-R at C9. For the rest of the carbon sequence, hyperbaric helium presents the highest Sn2 value in the Pro-S approach (Figure 7C); this tendency, however, is only clearly observed in the C10-C17 region of the Pro-R approach (Figure 7D). The hyperbaric argon’s Sn2 at C2-C9 generally matches the control 1 bar line, while the C10-C17 region is elevated in both Pro-R/Pro-S approaches and merges with the xenon 25 bar curve.

**Figure 8:**
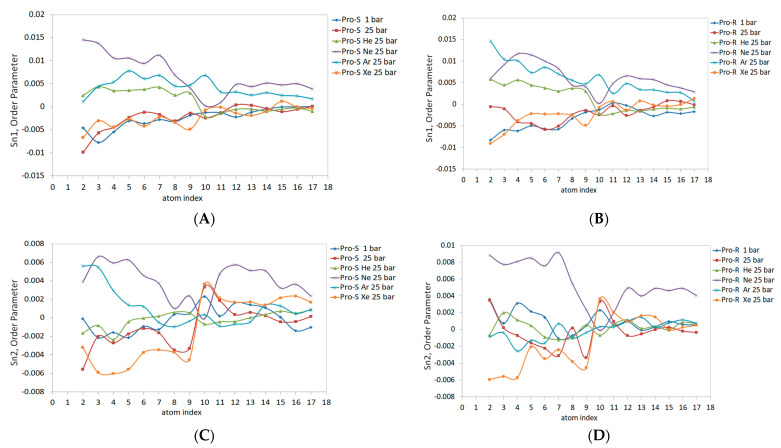
Internal monosphere DOPC acyl chains Sn1 and Sn2 prochiral order parameters: (**A**)—Sn1: Pro-S; (**B**)—Sn1: Pro-R; (**C**)—Sn2: Pro-S; and (**D**)—Sn2: Pro-R.

The DOPC inner spherical monolayer Sn1 and Sn2 order parameters are shown in Figure 8. Given the higher packing density of lipids in the inner monolayer, the Sn1 and Sn2 magnitudes generally shifted upscale compared to the outer monolayer values. The allocation of gaseous curves was adjusted as well; for example, the line attributed to neon at 25 bar maximizes Sn1 up to 0.015 for both Pro-S and Pro-R frames (Figure 8A,B). Unlike the outer monolayer case, the neon order parameter Sn1 curves display a clear positive concavity, with the same pattern of neon’s positively concaving curve observed in the Sn2 acyl chain (Figure 8C,D), although the maximal Sn2 values feature a lower (0.006) Pro-S and (0.008) Pro-R. Similar to neon, the xenon Sn1 and Sn2 lines differ significantly from the outer monolayer and represent the most declined trend in the C2-C10 region of Sn2 (Figure 8C,D). The Pro-S and Pro-R order parameters for Sn1 (Figure 8A,B) yield very similar data for hyperbaric xenon, hydrostatic pressure, and the control 1 bar simulation, despite the tendency of xenon atoms to specifically populate the inner monolayer, as presented in Figure 4D. The Sn1 lines for hyperbaric helium and hyperbaric argon (Figure 8A,B) represent closely scattered order parameter data, characterized by a raised argon line compared to the outer monolayer (Figure 7), along with opposite concavities—positive (He) vs. a negative argon line. The Sn2 curves for the helium–argon pairing at the Pro-R presentation (Figure 8D) coincide mostly with the control 1 bar line; the C1-C10 region, however, shows enhanced data variability, while the Pro-S argon line (Figure 8C) rises above the control 1 bar line, and the Pro-R argon line (Figure 8D) descends below the referential 1 bar curve.

After analyzing the order parameters in this study, we will address the order parameter considerations calculated from united-atom simulations, as Piggot et al. presented for the POPC molecule having an identical Sn2 oleoyl tail with the DOPC lipid [41]. The long simulations (t > 100 ns) for the planar bilayer with various united-atom force fields (e.g., GROMOS 43A1-S3 and the Berger force field in particular) result in the Pro-S and Pro-R order parameters noticeably splitting in the C2-C5 region, while the Pro-S of the hydrogen atom is larger than that of the Pro-R of the hydrogen atom (−0.153 and −0.163) [32]. Despite our data reporting significantly flattened order parameter curves, the Pro-S/Pro-R splitting at C2 (as it appears in Figure 7 and Figure 8) remains consistent with the POPC planar bilayer data. The double-bond C9-C10 atoms with an average value of 119.8° for the angles between their bisectional planes, calculated from the flat bilayer simulations, produce a characteristic dip at C10 on Sn curves. The dip is not observed at the control 1 bar simulation; however, it appears prominently in the outer monolayer saturated with helium at Sn1 and in the inner monolayer at Sn1 and Sn2 while saturated with neon at 25 bar. To summarize the above findings related to lipids’ order parameter, the patterns attributed to Sn variations inflicted by noble gases in planar bilayers generally increase the Sn value. At the same time, hydrostatic pressure might affect the ordering of lipids only at a very high magnitude exceeding 1000 bar [29]. The patterns can be reconsidered given the asymmetric 3D deformation of the liposome with local intra-bilayer inflations in restricted sectors by the continuous gas phase. While the lipid packing arrangement in the frustum shape causes a significant Sn deviation as has been reported also by Giupponi et al. [45], it preserves the fundamental structural properties of the planar bilayer that might be partially recovered by filling intermolecular voids with an inert hydrophobic diluent such as neon or helium.

## 4. Discussion

In our current research, we put the focus on the basic geometry refinement of the simulated system. Previously analyzing the embedded NMDA receptor, we have proven the planar membrane geometry with periodic boundary conditions to be inadequate for depicting the lateral wholeness of bubble nucleation, especially in the case of helium bubbles [32]. Considering lipid membrane finite closedness, explicit curvature and a sufficiently large volume of the bulk solvent merging with the lipids, the expansion of the basic geometry of the model has been expected to yield new insights into the molecular mechanisms of hyperbaric neurological disorders. Hence, the data analysis must not be limited to the bilayer and should be extended up to the water phase with respect to gaseous nanobubbles’ nucleation.

The large xenon nanobubble formed in the bulk solvent phase after 160 ns of MD time (Figure 5F) can be a simulation artifact or transient form with a limited lifespan, which demands further clarification. As relevant to our system, the reference model using the OPLS/TIP3F force field has been reported to depict free-floating inert gas (Xe, Kr) and diatomic nitrogen (N_2_) nanobubbles in 13 bar of ambient pressure, adsorbed to the globular protein surface and resulting in protein–gas aggregates [46]. It was speculated that such aggregates are loci of narcotic action, while protein’s enzymatic function is being notably impeded. The real morphology of narcotic gas—water solvent complexes—may imply nanostructured water–gas domains or clathrate-like blobs with neutral buoyancy that are not explicitly isolated from the water phase [47]. Combined coarse-grained (CG) and atomistic molecular dynamics (MD) simulations were performed to study the interactions of xenon with model lipid rafts consisting of 1,2-dipalmitoyl-sn-glycero-3-phosphocholine (DPPC), 1,2-dilauroyl-sn-glycero-3-phosphocholine (DLPC) and cholesterol (Chol) [48]; the spontaneous nucleation of Xe nanobubbles which rapidly plunged into the bilayer was observed assuming in such a scenario that the bilayer has not been preliminarily oversaturated.

The nucleation of xenon blobs or bubbles in the bulk solvent and the unidirectional diffusion of all dissolved gases into the hydrophobic bilayer cavity depend on the steady-state bulk gas concentration. This concentration, in turn, is defined by the magnitude of the energy barrier encountered by gas atoms at the interface between lipids and the bulk solvent [49]. The relatively high energy barrier at the xenon/lipid interface could potentially be re-estimated by considering the intrinsic polarizability of xenon, using force fields that account for such properties. The barrier could be notably pronounced in the case of charged lipids, considering the orientation of their headgroup [50]. However, intrinsic polarizability may contribute not only to a higher lipid interface barrier, defining xenon trapping with a headgroup region, which in turn defines the highest xenon lipophilicity amongst all noble gases under investigation, but also to more effective xenon atom–atom interactions in the bulk solvent [51]. In this regard, the nucleation and growth of xenon blobs/bubbles outside the bilayer should not be disregarded by polarizable force field approaches. The nonpolarizable Berger/SPC FF model we used might not adequately represent the quantitative parameters of gas diffusion. However, the explicit separation of xenon into lipid- and bulk solvent-associated gas phases (nanobubble) follows reported patterns of bubble appearance.

The condensed gas phase (nanobubbles) has a profound effect on the data reported in Figure 4, which appears to be in controversy with the lipophilicity series. This is because a larger dispersed xenon fraction must inherently occupy the lipid bilayer compared to gases with smaller atoms. However, the formation of neon and argon bubbles within the liposome, along with the significant xenon bubble in the bulk phase, markedly alters the well-known trend. The inner monosphere, characterized by sufficient lipid packing density, indeed preserves the original lipophilicity trend of noble gases during the percolation process. In the case of helium (Figure 4A) vs. neon (Figure 4B) within the first 20 ns, this is evident. However, as the neon bubble begins to grow in the interlayer gap, the trend reverses. More neon atoms become crowded in the bilayer void, causing them to leave the inner monolayer. Argon and xenon fractions in the inner monosphere are affected even more significantly by their condensed phase formation off the monospheres’ boundaries. In contrast, the xenon dispersed fraction is completely absorbed by the lipid phase, unlike other gases. Consequently, it can be concluded that the simulation model aligns with the known xenon lipophilicity feature when considering its scattered phase. The overall gas concentration in the system significantly exceeds the lipid phase saturation limits, which is the main cause for the alleged deviation of gas atom numbers from lipophilicity conduct, leading to subsequent separations of lipid monolayers [52]. A similar effect of excessive cholesterol concentration causing DOPC monolayers’ separation was reported, while CG simulations displayed the accumulation of cholesterol molecules at the interface of the lower and upper monolayers of the bilayer, thus leading to undulations in the bilayer with subsequent unzipping [53]. The real concentration of noble gases under hyperbaric conditions, or even clinical volatile anesthetics under standard conditions in the close microenvironment of the living neuron, is a matter of intensive debate. However, relatively high local gradients are possible at lipid boundaries, which makes nanobubble formation highly probable [54]. 

In the case of hyperbaric helium at a reduced concentration compared to the planar bilayer scenario, its hydrophobic effect does not surpass the energy cost of the enclosed monosphere’s decoupling and spatial separation. Consequently, the primary portion of the helium fraction in the system remains in the bulk solvent, distributed among a large number of nano-droplets. The anesthetic effect of nano-droplets obviously depends not only on their size (helium droplets’ volume does not exceed the size of a few united water molecules, less than 1 nm) but also on interactive stability. In several MD frames tested at MD time 90–100 ns, we observed a high mutability of droplet shapes and locations, which, in turn, can result in a very weak narcotic effect. Meanwhile, the maximum helium density peak, consistently attributed to the bilayer hydrophobic core, remains stable during the last 50 ns of the simulation. Assuming that the gas density peak in the bilayer core should be associated with CNS hyperexcitability, the tendency of neon and especially argon to cluster within the core to a much larger extent than helium seems to contradict such an assumption. This is noteworthy given the fact that argon is a strong anesthetic agent, and its hypoexcitable CNS effects are expected to strongly overcome the hyperexcitable ones [55]. 

The absence of explicit proteins in extramembrane domains (EMDs) in our system could be the determining factor for such an alleged discrepancy. The large hydrophobic voids within the extramembrane proteins can potentially divert a significant amount of argon from the bilayer core [56], triggering a strong competitive scenario between hypoexcitable and hyperexcitable segments, even within the same embedded protein complex responsible for electrochemical signal transduction. Gas interactions with hydrophobic voids vs. external molecular surfaces of the EMD can apparently distinguish between the bulk anesthetic effects of xenon and the invasive effects of argon atoms. These effects can be additive and alluding to Pauling’s theory of narcosis [57]. 

The next important question that must be raised is how well a small liposome with sharp membrane curvature represents the physiological processes and the closedness of a whole-cell-like lipid membrane. Membrane curvature is a crucial characteristic because the exchange of molecules and signals across the lipid barrier in living cells involves various processes, such as the bending, invagination, fusion and unzipping of the membranes. The curvature of the membrane is triggered by modifications in the local lipid composition, whether it involves the lipid headgroup, tail or cholesterol enrichment [58,59]. Similar to cholesterol, the oversaturation of the naturally highly heterogeneous lipid phase by noble gases may upset the delicate balance between lateral forces within the outer and inner monospheres, causing either positive curvature (towards the cytoplasm) or negative curvature (away from the cytoplasm) to appear. The lateral pressure profile existing in the planar bilayer due to very intense packing density can reach high quantities, up to 600 bar (positive pressure) in the headgroup regions and down to −200 bar (negative pressure) in the lipid tails’ domain. One of the well-established theories of the lipid mechanism of general anesthesia by Cantor suggests that the lateral pressure profile is significantly flattened by clinical GA with effectively long carbon backbones [60]. However, this is not the case for scattered xenon atoms, as reported by Booker et al. [61]. They showed the inability of xenon to dramatically change the lateral pressure profile in the DOPC planar bilayer, despite its known tendency to populate the lipid headgroup domain and cause a considerable increase in area per lipid. We imply that the inflation of the bilayer void by the condensed gas phase might cause a significant local imbalance between lateral forces in the outer vs. inner monolayers. This may supply the resulting force for further interlayer void growth without perturbing the general lateral pressure profile per se.

The synaptic vesicle (SV) serves as a firm example of a molecular system in neurological signal transduction, where the explicit curvature of the lipid bilayer plays a crucial role. Neuronal transmitters are packaged in SVs, which are released from the neuron’s presynaptic membrane into the synaptic gap [62]. Computational research using the MD method has also investigated SV release. The CHARMM36/TIP3P water force field model with a lipid vesicle radius set to 11 nm, identical to the current liposome model, has been employed in these studies [63]. The SV’s exocytosis is heavily regulated by protein complexes, but the process has been determined to be directly dependent on the contact area between the surfaces of SVs and plasmatic membranes. Precise molecular simulations of hyperbaric gases’ effects on presynaptic release are needed. However, we can speculate that considerable liposome shape distortion and an increase in the surface of the outer monolayer per se might induce the formation of extended membrane–membrane contact interfaces and have a considerable impact on the kinetics of the membrane fusion process.

Summarizing the presentation of our data, one should be consistent with the fact that noble gases cannot have obvious biomolecular specificity like other neuromodulators and therefore their effects on the CNS are amenable to some statistical generalization, as suggest the hypotheses concerning the molecular effects of general anesthetics. Considering the entire set of molecular events described in our work—in particular, the clear separation of continuous hydrophobic gas fractions into the condensed xenon fraction associated with the bulk solution and the fractions of gases confined in a lipid medium, and the growing body of evidence that neither lipid nor protein theories of general anesthesia can perfectly cover available experimental and theoretical evidence of nobles gases’ neurological effects—we tend to support the tenets of Halsey et al. [64] in their presentation of the multisite expansion theory of GA action—‘1. General anesthesia can be produced by the expansion of more than one molecular site with different physical properties; 2. The physical properties of a particular site can be influenced by anesthetics and pressure, 3. The molecular site or sites do not behave as bulk solvents but have finite sizes and limited occupancy.’

As shown in our simulations, the nanobubble formation at various loci in the immediate vicinity of the lipid membrane complies with the multisite expansion theory’s propositions, which can be further reformulated since not all hydrophobic expansions (distortions) can be clearly associated with inert gas anesthesia but also with opposite phenomena—helium tremors and the explicit hyperexcitability of the CNS. The fact that anesthetic argon and a fraction of xenon steadily percolate the lipid phase is evidence that anesthetic sites can be multifocal and span large distances; they may be immersed in the lipid boundary or fully exposed to the bulk solution. This indicates that non-immobilizing neon and helium can compete for the same molecular sites as argon and xenon, while the all-encompassing effect on the conformational dynamics of embedded channels should be considered by small gas atoms vs. large and considerably polarizable atoms.

Additional information on the possible neurological effect of condensed gas fractions associated with biomolecular complexes is available from the previous publications. For instance, a xenon nanobubble in the lumen of ion channels has been suggested by Roth et al. [65] as part of the molecular mechanism of narcosis resulting in the blockade of ion transport. However, their approach does not consider the dynamics of ion channel configuration or the possibility of gases diffusing through the channels’ lumen. In this regard, hydrophobic gating in the inner pore of a channel caused by hydrophobic residues seems more realistic as a potential target of inert gas action; it can be further intensified by continuous gas bubble formation or reduced by uniformly scattered GA [66]. Different solvation patterns of the primary ions should be meticulously considered when analyzing liquid–vapor transitions within the pore, although different ions are not uniformly vulnerable to this effect [67]. As a molecular site where affected bulk properties of the bilayer may translate to specific embedded protein responses, inositol functional groups (PIP2), for example, were denoted that might control HCN^−^ and activate Ca^2+^ channels [68]. Lipid tail inclusions in “fenestrations” at the interface between the transmembrane helices in channels that potentially expose the ion conduction pathway to the lipid core of the bilayer can also be modulated by HP/GA [69,70]. Regarding the diffusive nature of the helium phase, it can inflict molecular distortions alongside conformational uncertainty and likely impedes precise docking interactions for guided ligand molecules in the extramembrane protein domain [71]. Meanwhile, enhanced hydrophobic gating in the lipid membrane saturated with helium might be an additional cause for the induced hyperexcitability. The molecular surface of the extramembrane domain of large proteins such as NMDAR, conversely, can be targeted by free-floating xenon nanobubbles, while the resulting gaseous-protein complex will present significantly deferred conformational dynamics and consequently impair the transmembrane flow in an opposite manner.

## 5. Conclusions

The small lipid vesicle differs substantially from the planar bilayer and is characterized by significant structural asymmetry quantified by independent order parameter measurements. This membrane undergoes significant native shape distortion due to hyperbaric argon and neon, with the bubble nucleation in the inter-bilayer gap causing the significant deformation of the original spheroid boundary. The pressure coupling algorithms have no explicit effect on the bubble nucleation patterns, which apparently depend on the local gas concentration gradients and solvent inhomogeneity at the nanoscale. Hyperbaric helium serves as an effective pressure transducer accumulated in the hydrophobic lipid core as a uniformly scattered fraction but not as a continuous bubble (contrary to the previously reported planar bilayer case). Hyperbaric xenon consistently forms the second type of bubble located exclusively in the bulk solvent, while its lipid fraction presents as uniformly scattered atoms. Assuming the extramembrane protein domains protrude to significant distances, their association with large xenon bubbles suppresses electrochemical signal transduction. A fraction of helium and neon remains at the bulk solvent in the form of small droplets that might induce similar residual anesthetic effects. Hyperbaric argon forms the largest continuous bubble in the hydrophobic void, given its mostly anesthetic nature—the conclusion can be drawn that the same hydrophobic locus, either in the lipid membrane or in the extramembrane protein domain, can accommodate both excitatory and inhibitory macromolecular fragments. The resulting central nervous system conditioning is obviously defined by the dynamic balance of all loci’s interplay. The nanobubble’s nucleation and expansion, as consistently shown in computational experiments, strongly support the multisite expansion (distortion) theory of general anesthetic action.

## Figures and Tables

**Table 1:** The liposome dimensionless data at 25 bar pressure with different gases normalized to 1 bar* values.

Simulation	Volume [Å^3^]	Accessible Surface Area [Å^2^]	Sphericity Index	Effective Radius [Å]
1 bar*	2,429,241	193,770	0.45	37.61
25 bar	0.997	0.998	1	0.999
He, 25 bar	1.025	1.025	1	0.999
Ne, 25 bar	1.029	1.356	0.756	0.759
Ar, 25 bar	1.034	1.722	0.6	0.601
Xe, 25 bar	1.027	1.056	0.956	0.956

**Table 2:** The dimensionless gas phase molecular volumes associated with the liposome, normalized by the volume of continuous gas fractions in the external bulk solvent * (second left column).

Simulation	External Bulk Solvent * [Å^3^]	Outer Monosphere	Interlayer Gap	Inner Monosphere	Confined Solvent
He 25 bar	31,630	0.226	0.037	0.385	0.032
Ne 25 bar	30,852	1.467	2.982	1.183	0.005
Ar 25 bar	11,595	6.979	14.877	5.677	0.013
Xe 25 bar	471,022	0	0	0.0001	0

## Data Availability

The original contributions presented in the study are included in the article; further inquiries can be directed to the corresponding authors.

## Supplementary Materials

The following supporting information can be downloaded at https://www.mdpi.com/article/10.3390/membranes14040089/s1, Figure S1: Isotropic pressure coupling simulations (t = 50 ns, last 10 ns have been used for density analysis). (A) DOPC lipids density at 1 bar with various molar concentrations of dissolved argon: 0%; 0,375%; 0,750% and 1.5%; (B) The dissolved argon at 1 bar with various molar concentrations: 0,375%, 0,750% and 1.5%; The liposome’s center of mass located at x = 0 at the origin of the x axis, Figure S2: Isotropic pressure coupling simulations. (A) DOPC lipids density at 25 bar with 1.5% argon concentrations, MD frames: t = 0, 20, 100 ns; (B) The dissolved argon density at 25 bar with 1.5% concentrations, MD frames: t = 0, 20, 100 ns. The liposome’s center of mass located at x = 0 at the origin of the x axis, Figure S3: Isotropic pressure coupling simulation. Argon atoms counter is presented at 25 bar for 5 compartments of the simulation box vs. MD time (100 ns), Figure S4: Semi-Isotropic Pressure coupling. Representative MD trajectory frames of DOPC liposome at 1 bar; at t = 0, 100 ns, Figure S5: Semi-Isotropic Pressure Coupling. Representative MD trajectory frames of DOPC liposome at 25 bar; at t = 0, 100 ns, Figure S6: Semi-Isotropic Pressure Coupling. Representative MD trajectory frames of DOPC liposome and dissolved helium at 25 bar; MD frames are shown at t = 0, 20, 100 ns, Figure S7: Semi-Isotropic Pressure Coupling. Representative MD trajectory frames of DOPC liposome and dissolved neon at 25 bar; MD frames are shown at t = 0, 20, 40, 100 ns, Figure S8: Semi-Isotropic Pressure Coupling. Representative MD trajectory frames of DOPC liposome and dissolved argon at 25 bar; MD frames are shown at t = 0, 20, 100 ns, Figure S9: Semi-Isotropic Pressure Coupling. Representative MD trajectory frames of DOPC liposome and dissolved xenon at 25 bar; MD frames are shown at t = 0, 20, 40, 60, 80, 100, 120, 140, 160 ns.
