# Supplementary material for: Studying the Effects of Dissolved Noble Gases and High Hydrostatic Pressure on the Spherical DOPC Bilayer Using Molecular Dynamic Simulations
Source: Membranes (Basel). 2024 Apr 12;14(4):89. doi: 10.3390/membranes14040089 (PMC11052037; doi:10.3390/membranes14040089)
Supplement: Supplementary file 1 [file membranes-14-00089-s001.zip › membranes-2896105-supplementary.pdf]

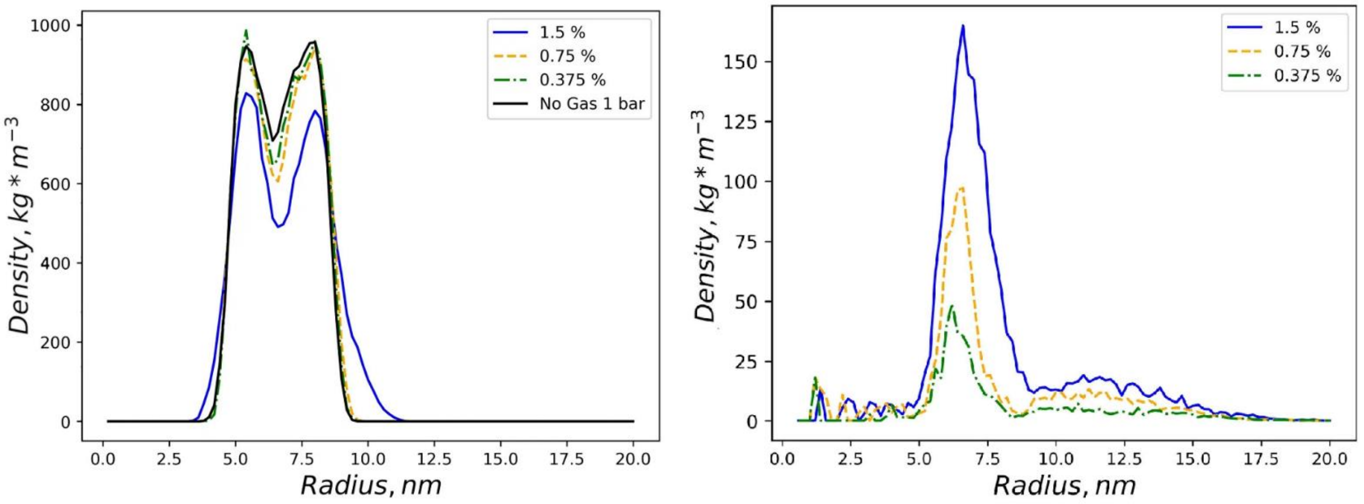

(A) **(B)**

Figure S1. Isotropic pressure coupling simulations (t = 50 ns, last 10ns have been used for density analysis). **(A)** DOPC lipids density at 1 bar with various molar concentrations of dissolved argon: 0%; 0,375%; 0,750% and 1.5%; **(B)** The dissolved argon at 1 bar with various molar concentrations: 0,375%, 0,750% and 1.5%; The liposome's center of mass located at x = 0 at the origin of the x axis. .

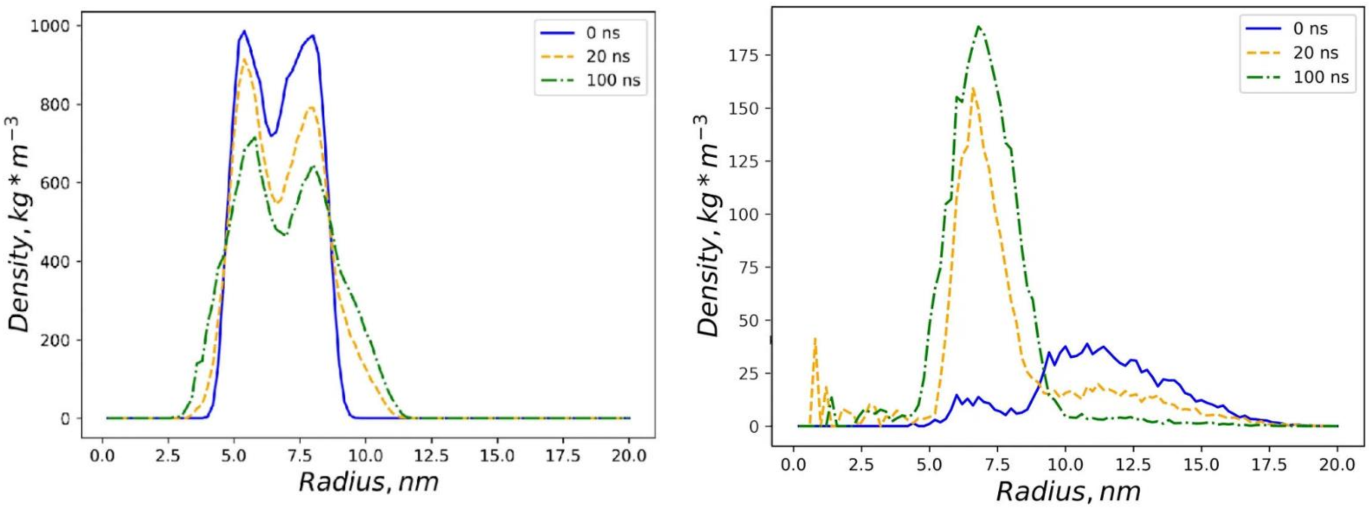

(**A**) (**B**)

Figure S2. Isotropic pressure coupling simulations. **(A)** DOPC lipids density at 25 bar with 1.5% argon concentrations, MD frames: t = 0, 20, 100 ns; **(B)** The dissolved argon density at 25 bar with 1.5% concentrations, MD frames: t = 0, 20, 100ns. The liposome's center of mass located at x = 0 at the origin of the x axis.

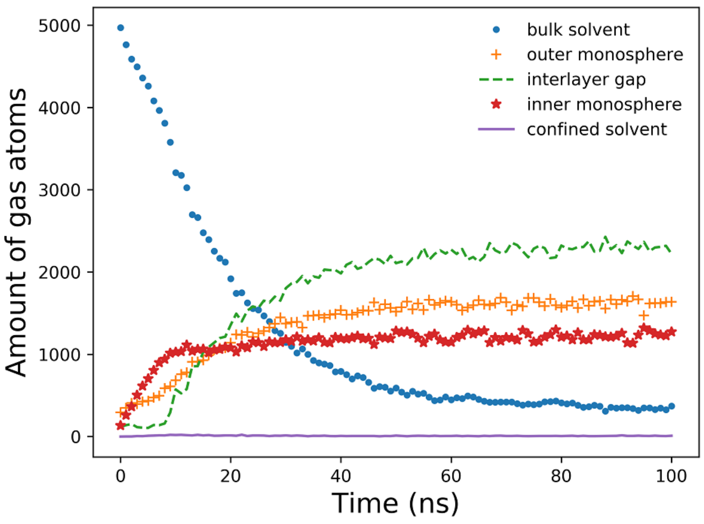

Figure S3. Isotropic pressure coupling simulation. Argon atoms counter is presented at 25 bar for 5 compartments of the simulation box vs. MD time (100 ns).

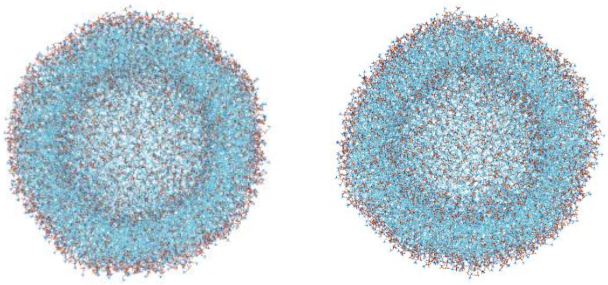

No gas 1 bar, 0 ns **No gas 1 bar, 100 ns**

Figure S4. Semi-Isotropic Pressure coupling. Representative MD trajectory frames of DOPC liposome at 1bar; at t= 0, 100 ns.

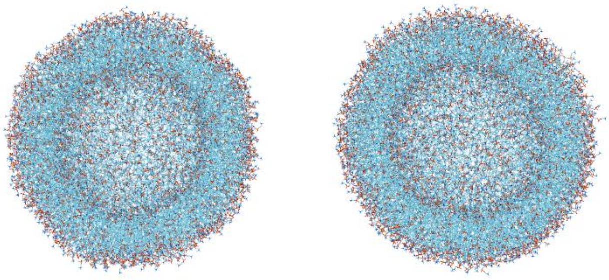

No gas 25 bar, 0 ns **No gas 25 bar, 100 ns**

Figure S5. Semi-Isotropic Pressure Coupling. Representative MD trajectory frames of DOPC liposome at 25bar; at t= 0, 100 ns.

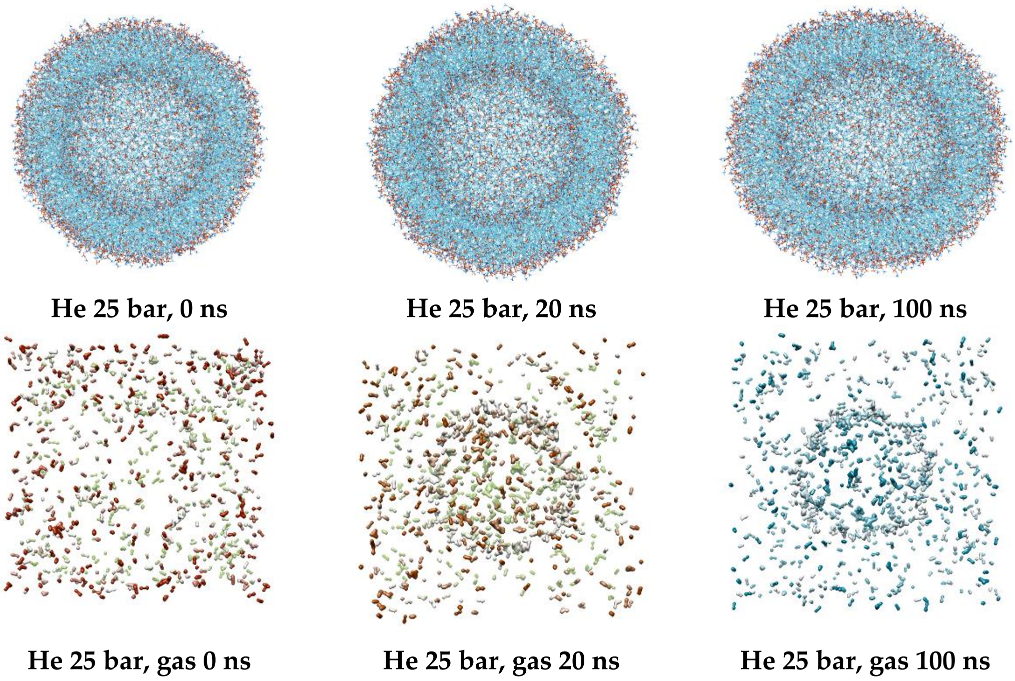

Figure S6. Semi-Isotropic Pressure Coupling. Representative MD trajectory frames of DOPC liposome and dissolved helium at 25bar; MD frames are shown at t= 0, 20, 100 ns.

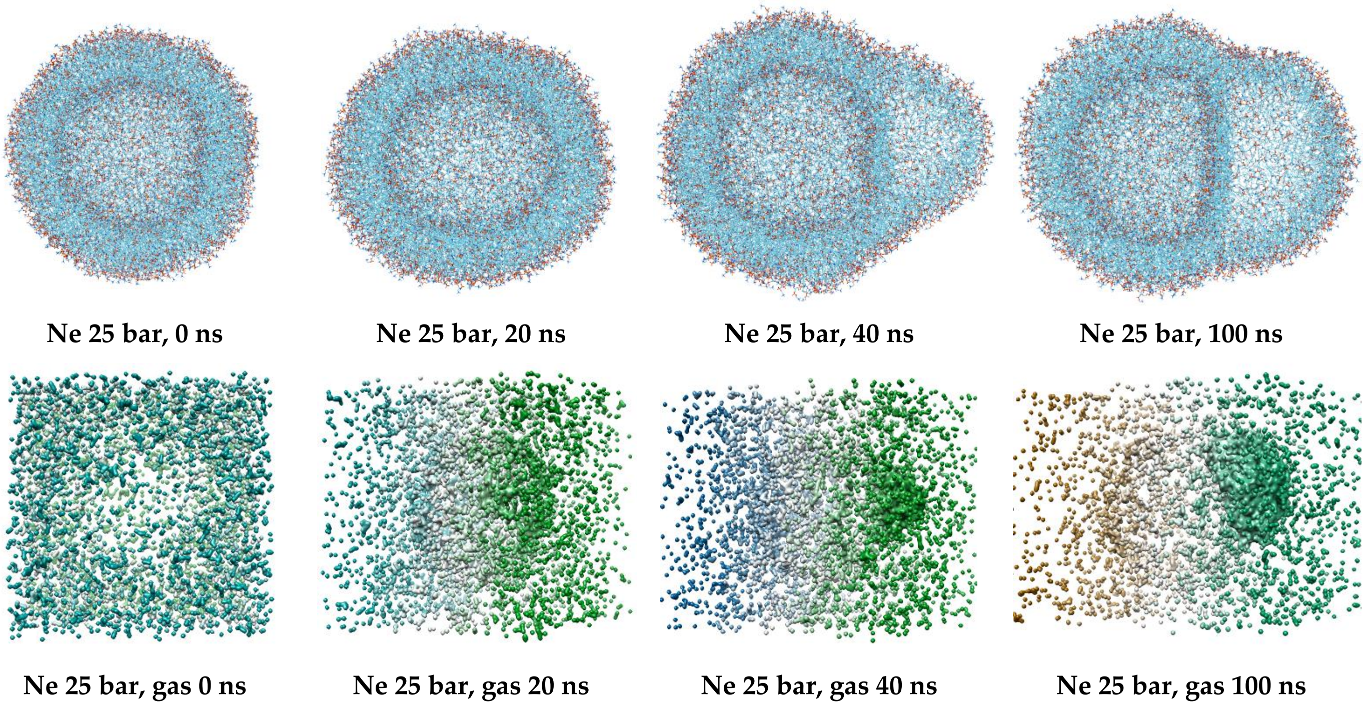

Figure S7. Semi-Isotropic Pressure Coupling. Representative MD trajectory frames of DOPC liposome and dissolved neon at 25bar; MD frames are shown at t = 0, 20, 40, 100 ns.

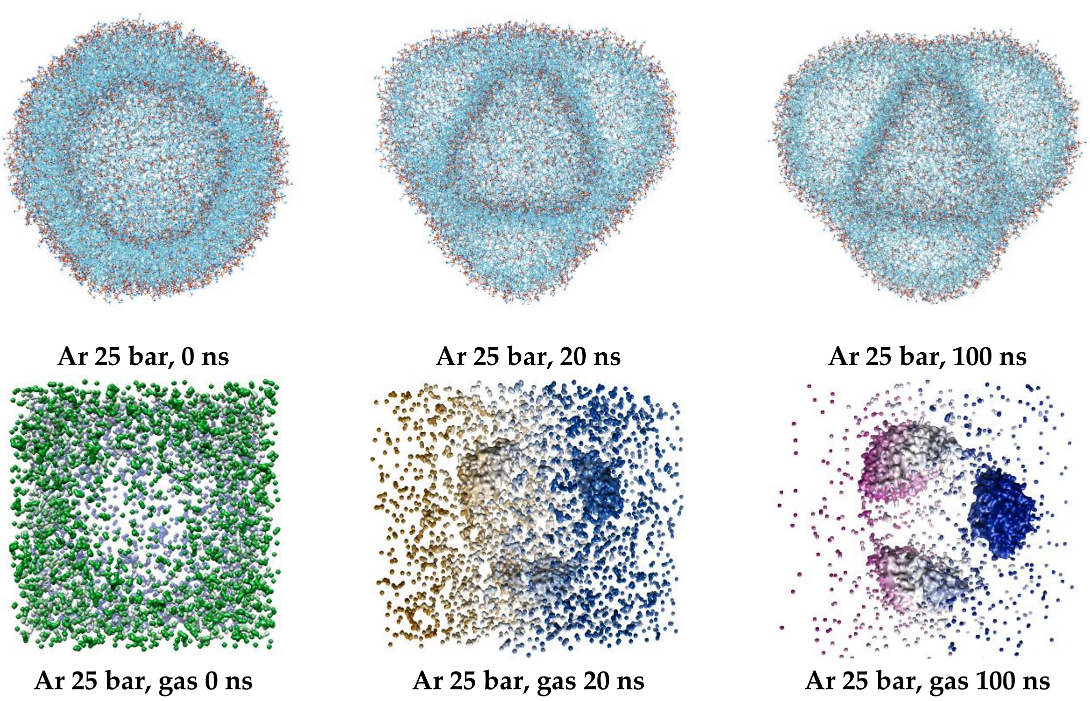

Figure S8. Semi-Isotropic Pressure Coupling. Representative MD trajectory frames of DOPC liposome and dissolved argon at 25bar; MD frames are shown at t = 0, 20, 100 ns.

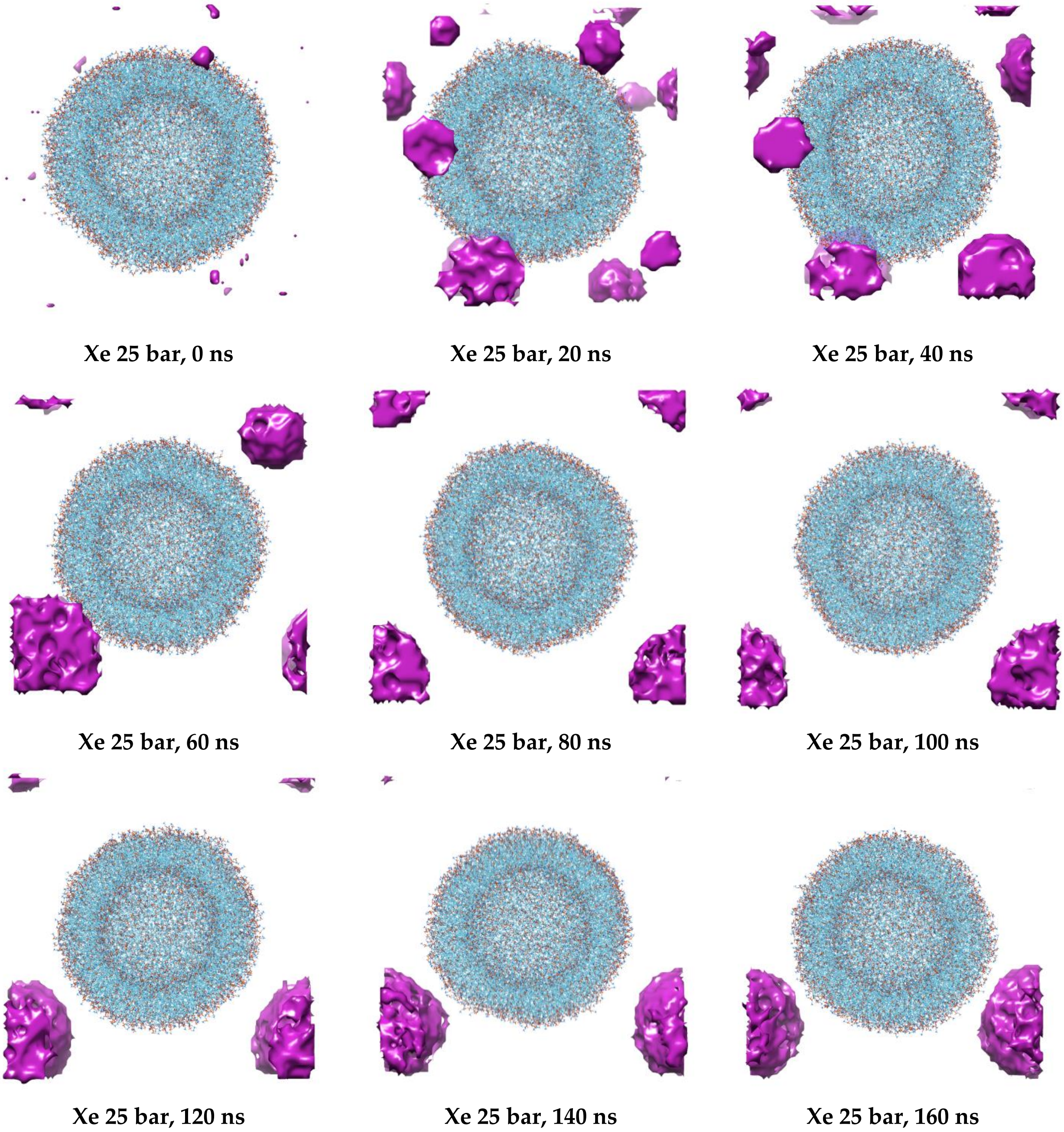

Figure S9. Semi-Isotropic Pressure Coupling. Representative MD trajectory frames of DOPC liposome and dissolved xenon at 25bar; MD frames are shown at t = 0, 20, 40, 60, 80, 100, 120, 140, 160 ns.
